# Stratification of patients with autoinflammatory phenotypes by interferon (IFN) score suggests a new group of IFN mediated autoinflammatory diseases with overlapping clinical phenotypes

**DOI:** 10.1186/1546-0096-13-S1-O35

**Published:** 2015-09-28

**Authors:** A Almeida de Jesus, Z Deng, S Brooks, H Kim, G Montealegre, D Chapelle, Y Liu, B Marrero, L Malle, M O'Brien, W Goodspeed, Y Huang, P Hashkes, G Nasrullayeva, MT Terreri, C Silva, B Arabshahi, K O'Neill, M Punaro, L Moorthy, A Reinhardt, V Lilleby, J Niemela, S Rosenzweig, T Fleisher, R Goldbach-Mansky

**Affiliations:** 1 National Institutes of Health, Translational Autoinflammatory Diseases Section, Bethesda, USA; 2 National Institutes of Health, NIAMS, Bethesda, USA; 3 Hebrew University School of Medicine, Jerusalem, Israel; 4 Azerbaijan Medical University, Department of Immunology, Baku, Azerbaijan; 5 Federal University of Sao Paulo, Sao Paulo, Brazil; 6 University of Sao Paulo, Sao Paulo, Brazil; 7 Inova Fairfax Hospital, Fairfax, USA; 8 Riley Children's Hospital, Indianapolis, USA; 9 Children's Medical Center Dallas, Dallas, USA; 10 Robert Wood Johnson University Hospital, New Brunswick, USA; 11 University of Nebraska Medical Center, Omaha, USA; 12 Rikshospitalet University Hospital, Oslo, Norway; 13 National Institutes of Health, Department of Laboratory Medicine, Bethesda, USA

## Background

We have identified mutations in proteasome components as the cause of CANDLE syndrome and in *TMEM173/STING* as the cause for a severe vasculopathy and lung disease, SAVI. CANDLE and SAVI patients do not respond to IL-1 inhibition and consistently demonstrate marked up-regulation of IFN-inducible genes. Our data suggest innate immune dysregulation caused by chronic Type I IFN signaling in both conditions.

## Objective

We hypothesize that the presence of IFN signature may identify patients with autoinflammatory disease (AID) who have genetic mutations in other IFN regulating genes.

## Methods

To identify patients with IFN signatures, RNA sequencing (RNA-seq) from whole blood RNA was performed using HiSeq 2000 Illumina^®^ platform. Heatmaps with 64 IFN response genes were assessed. Whole exome sequencing (WES) was performed from whole blood DNA.

## Results

We identified 19 patients with marked upregulation of IFN inducible genes. WES was performed in 14 patients and parents (trios) and in 5 individual patients. Of the probands, 9/19 were female, 8/19 were Caucasian, 3 Asian, 2 Hispanic, 2 Norwegian and 4 had other ethnicities. All patients presented with immunodysregulatory phenotypes with clinical similarities to the previously described interferonopathies, including skin vasculitis/vasculopathy (9/19), panniculitis (12/19), myositis (5/19) and basal ganglion calcifications (5/19), but had no genetic diagnosis prior to NIH evaluation. The bioinformatics variant annotation, analysis and filtering workflow successfully identified mutations in IFN-regulating genes in 7 of the 19 probands. In one patient, we found a disease causing *de novo* and somatic mutation in *TREX1*. This patient also presented with an in-frame deletion in *DHX9* inherited from her mother and a missense mutation in *MAVS* inherited from her father. In one patient, we identified a *de novo* mutation in *DHX9* and this patient is also a compound heterozygous for mutations in *IFIH1/MDA5*. In a third patient, we found a missense mutation in *TREX1* inherited from the mother and a heterozygous variant in *MB21D1* (gene encoding cGAS) inherited from the father. A fourth patient with a clinical phenotype of CANDLE had two novel compound heterozygous mutations in *PSMG2*. Additionally, a male patient with lupus-like clinical and laboratory findings was found to have an X-linked mutation in *TREX2* gene. All mutations described were confirmed by Sanger sequencing.

## Conclusion

RNA-seq can be a tool for the identification of patients with an IFN signature and guide the search for disease causing variants in IFN-regulating genes by WES. However, disease causality of these mutations needs to be assessed in functional assays. Moreover the identification of patients with a type I interferon signature and a set of clinical features that are not seen in IL-1- mediated-AIDs allow stratification of a subset of AIDs that are typically “poor IL-1 responsive”. Whether the IFN signature identifies a subset of patients that respond to the blockade of Type I IFN signaling needs to be further validated.

